# Dental esthetic screening index (DESI) scores among patients attending Qassim University dental clinics

**DOI:** 10.1016/j.sdentj.2024.04.007

**Published:** 2024-05-01

**Authors:** Norah M Almufadhi, Ola.M. Sakr, Lina Aloufi

**Affiliations:** a General Dental Practitioner, Qassim Armed Force Hospital, Buraydah, Qassim 52558, Saudi Arabia; b Department of Conservative Dentistry, College of Dentistry, Qassim University, Buraydah, Qassim 52571, Saudi Arabia; c Department of Operative Dentistry, College of Dentistry, Misr University for Science and Technology, Egypt

**Keywords:** Dental esthetic screening index (DESI), Dentofacial esthetics, Restorative dentistry

## Abstract

**Aim:**

This study aimed to evaluate dental esthetic screening index (DESI) Intraoral component scores among patients attending Qassim University dental clinics and the factors affecting these scores.

**Materials and methods:**

This was a retrospective study that included 150 participants aged 18 years or older with full upper and lower anterior dentitions. Standardized intraoral frontal photographs of the patients’ upper and lower dentitions were acquired from their dental records, and the mean rank DESI intraoral scores were evaluated and compared across the demographic and dental characteristics of the study patients.

The mean age of the patients sampled was 33.56; 57 % were female and 43 % were male. The mean DESI Intraoral score was 15.33. DESI scores were significantly higher in patients older than 35 years compared with subjects 35 years old or younger (*p* = 0.015). DESI scores were increased in patients who had anterior restorations compared with those who did not (*p* = 0.016).

**Conclusions:**

This was the first study of its kind to use the DESI in a large clinical setting. According to the DESI, most patients attending Qassim University dental clinics had good dentofacial aesthetics. Age and the presence of anterior restorations negatively impacted patients' dentofacial aesthetics. Sex, the nature of the chief complaint, a history of orthodontic treatments, and teeth bleaching did not affect the DESI scores.

## 1 Introduction

The perception of beauty, in general, is subjective and is influenced by variable factors such as evidence-based literature, cultural differences, and civilizations (Abu Alhaija et al. (2005)). The perception of dentofacial aesthetics is influenced by individuals' experiences and social backgrounds (Flores-Mir et al. (2004)).

A vast amount of literature tries to standardize or create principles of cosmetic smiles by managing the effects of lip framework, gingival tissue, and dentition (Sharma and Sharma (2012)). Dental professionals should make an effort to explore and understand the esthetic view of people regarding the elements of smile esthetics so that they do not unnecessarily treat small changes that go unrecognized by the patient, especially since the general population is less receptive to minimal deviations from the ideal parameters (Machado et al. (2013)). Such treatments often require a multidisciplinary team that requires different specialists, time, and money. This understanding would help improve communication and assist in determining a common treatment objective (Pinzan-Vercelino et al. (2020)).

Previous studies have shown that dental specialists and patients have different opinions about the perception of smile attractiveness (Roden-Johnson et al., 2005, Katiyar et al., 2016). Additionally, the assessments of dentofacial esthetics differ considerably among dental specialists in different dentistry fields (Prica et al. (2022)).

Measuring dentofacial esthetics is challenging, and there is a need for a comprehensive index that can achieve this task (Frese et al., 2012, Rosenstiel et al., 2009). Since the early 1970 s, the Modified United States Public Health Service (USPHS) criteria or the Fédération Dentaire Internationale (FDI) clinical criteria have been the most widely used clinical evaluation tools in restorative dentistry (Hickel et al., 2007, Hickel et al., 2010). However, these criteria provide inadequate detail for a complete baseline, outcome assessment of restorative treatment options for the anterior teeth, and the impact of the treatment on the overall dentofacial appearance (e.g., smile assessment) (Frese et al. (2012)).

The Prosthetic Esthetic Index (PEI) was designed to assess the dentofacial esthetics of patients receiving prosthetic treatments (Özhayat and Dannemand (2014)). This index is an objective tool for clinical diagnosis, analysis, and treatment planning. However, it cannot be used to quantify the overall dentofacial appearance since it cannot assess intraoral soft and hard tissues. As a result, the Dental Esthetic Screening Index (DESI) was created in 2019. This index includes three extraoral and seven intraoral components. The DESI assesses quantitative data for each item using a five-point rating scale and categorizes the esthetic outcome using a cumulative score. A low value indicates good aesthetics, whereas a large value denotes poor aesthetics (Frese et al. (2019)).

The newly designed DESI is a valid and reliable tool to quantify the clinical condition of the patient and treatment outcomes in line with the patient's subjective perceptions. However, future research should investigate the sensitivity and specificity of this novel tool in various contexts to demonstrate whether an esthetic smile can be accurately measured (Frese et al. (2019)).

This study aimed to evaluate DESI intraoral component scores among patients attending Qassim University dental clinics and the factors affecting these scores.

## 2 Methods and Materials

This study was approved by the Institutional Review Board at Qassim University, College of Dentistry (Ethical approval number: F-2018–3003). All patients in this study provided written informed consent for their photos to be used for research purposes.

This was a retrospective study conducted in Qassim University dental clinics. Study participants were patients attending Qassim University dental clinics aged 18 years or older with full upper and lower anterior dentitions. Patients younger than 18 years old, patients with extracted anterior teeth due to any pathology, or patients with congenitally missing or impacted anterior teeth were excluded.

The sampling method used was a simple random sample from patient records, and the minimum sample size was 100 patients. The sample size was calculated at an α power of 80 %, and the α error type was set at a 0.05 significance level.

The data collected included patients' demographic information (age, nature of chief complaint, and dental and medical histories), standardized intraoral images before starting treatment at Qassim University dental clinics, and DESI intraoral component scores. Data were collected from new patient records from August 2019 to March 2020.

Seven components were used to calculate the DESI intraoral score, using a five-point scoring system that enabled progressive grading of aesthetic deviations. To quantify the aesthetic deviations, a measuring scale with numerical subdivisions was used. The sums of the intraoral components were between 7 and 35 points; a low total score indicated excellent esthetics, while a large sum score signified poor esthetics (Frese et al. (2019)). The clinical recording of the patients’ scores was done by a single examiner and was not repeated.

Data were analyzed using IBM SPSS Statistical software for Windows version 26.0 (IBM Corp., Armonk, N.Y., USA). Descriptive statistics were used to describe the quantitative and categorical variables. The DESI score values were not normally distributed, as the *p*-values of both normality tests (Kolmogorov-Smirnov and Shapiro-Wilk) were less than 0.05 (0.001, 0.001, respectively); hence, non-parametric statistical tests (Mann-Whitney *U* test and Kruskal-Wallis test) were used to compare the mean ranks of the DESI scores across the categorical study variables, including age, sex, chief complaint, history of teeth bleaching, history of orthodontic treatments, and presence of direct or indirect anterior restorations. The significance of all the statistical tests was set at a *p*-value less than or equal to 0.05.

## 3 Results

This study included 150 patients. The mean age of the participants was 33.56, with a standard deviation of 11.14; 57 % were female, while 43 % were male. The chief complaints were categorized into four categories: routine check-ups and teeth cleanings (3 %), restoring teeth and replacing missing teeth (61 %), pain and swelling (29 %), and dentofacial esthetics (6 %). Only 1 % of the patients had a history of teeth bleaching, 12 % received orthodontic treatments, and 45 % had direct or indirect restorations in their anterior dentition (Table 1).

**Table 1:** Distribution of demographic and dental care variables of study subjects (n = 150).

Variables	No. (%)
Age groups (in years)	
<=35	98 (65.3)
>35	52 (34.7)
Gender	
Male	65 (43.3)
Female	85 (56.7)
Chief complaint	
Related to routine check-up/cleaning teeth	6 (4.0)
Related to restoring teeth/replacing missing teeth	92 (61.3)
Related to pain/swelling	43 (28.7)
Related to dentofacial aesthetics	9 (6.0)
History of teeth bleaching	
Yes	1 (0.7)
No	149 (99.3)
History of orthodontic treatment	
Yes	13 (8.7)
No	137 (91.3)
Presence of direct or indirect anterior restorations	
Yes	68 (45.3)
No	82 (54.7)

**Graph 1:**
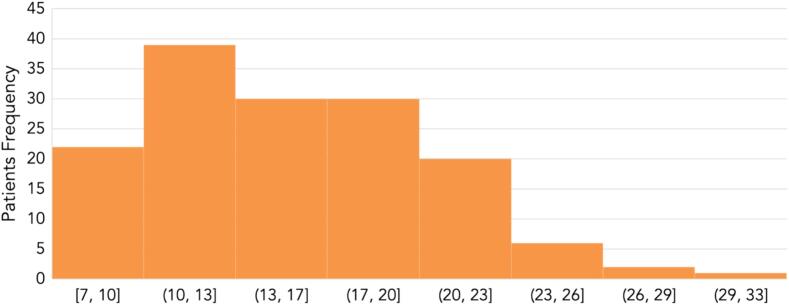
Dental esthetics screening index (DESI) intra-oral component scores among sampled patients.

The mean DESI intraoral component score was 15.33, with a standard deviation of 4.87. According to the index, the participants' scores were divided into five categories: excellent, good, satisfactory, insufficient, and poor esthetics, with most patients (82 %) being in the good esthetics category (Table 2) (Graph 1).

**Table 2:** Distribution of Dental Esthetic Screening index (DESI) intra-oral component score categories.

Categories	No. (%)
Excellent Esthetics	5 (3.3)
Good Esthetics	123 (82.0)
Satisfactory Esthetics	19 (12.7)
Insufficient Esthetics	3 (2.0)
Poor Esthetics	-

The comparison of the mean rank DESI scores across the demographic and dental characteristics showed a statistically significant difference in the DESI scores in relation to age and the presence of direct or indirect anterior restorations. The DESI scores were significantly higher in patients 35 years or older compared with subjects younger than 35 (*p* = 0.015).

The DESI scores were higher in patients with a history of direct or indirect anterior restorations compared with patients without restorations (*p* = 0.016). However, no statistically significant differences were observed in the mean ranks for the DESI scores related to the categorical variables of sex, chief complaint, history of teeth bleaching, and orthodontic treatments (Table 3).

**Table 3:** Comparison of mean ranks of DESI scores across the demographic and dental characteristics of study subjects.

Variables	Mean ranks of DESI scores	Mann-Whitney/Kruskal-WallisTest-statistic	*p*-value
Age groups (in years)			
<=35	69.26	1936.00	0.015*
>35	87.27		
Gender			
Male	78.71	2554.00	0.428
Female	73.05		
Chief complaint			
Related to routine check-up/cleaning teeth	51.67	6.720	0.081
Related to restoring teeth/replacing missing teeth	71.72		
Related to pain/swelling	81.30		
Related to dentofacial aesthetics	102.28		
History of teeth bleaching			
Yes	42.0	41.00	0.560
No	75.72		
History of orthodontic treatment			
Yes	60.69	698.00	0.197
No	76.91		
Presence of direct or indirect anterior restorations			
Yes	84.84	2153.00	0.016*
No	67.76		

* Statistically significant.

## 4 Discussion

Dentofacial aesthetics are subjective, but there have been multiple indices to measure and quantify them for clinical evaluation. The DESI is a novel tool to screen and examine changes in dentofacial esthetics before and after treatments. This study used the DESI to examine the dentofacial aesthetics of new patients at Qassim University dental clinics.

Various clinical measurement tools are used to evaluate dentofacial aesthetics in restorative dentistry. The Smile Esthetic Index (SEI) covers certain prosthodontic aesthetic elements within the broader assessment of smile aesthetics (Rotundo et al. (2015)). While this tool demonstrates strong inter-rater and intra-rater reliability, it has not gained wide use in research or clinical day-to-day practice (Mursid et al. (2020)).

The PEI is an esthetic assessment tool that has undergone validation and reliability testing in prosthodontic cases and has exhibited good responsiveness (Özhayat and Dannemand, 2014, Øzhayat, 2017). One major flaw of the PEI is that it cannot be employed to quantify the overall appearance of the teeth and face (Özhayat and Dannemand (2014)).

Furhauser et al. proposed the Pink Esthetic Score (PES) index to evaluate oral soft tissue aesthetics. Studies have shown that the PES could be a vital tool for monitoring soft tissue changes over an extended period (Fürhauser et al. (2005)). The White Esthetic Score (WES) index was later introduced by Belser et al. to exclusively focus on oral hard tissue aesthetics and evaluate tooth surface discrepancies (Belser et al. (2009)). Other studies have shown that these indices are reliable and have reproducible scoring methods (Tettamanti et al. (2016)).

The most recent index is the DESI, established by Frese et al. (2019), building upon their earlier review findings from 2012. The DESI has been proven to be a reliable and valid tool for quantitatively assessing dentofacial aesthetics (Frese et al., 2012, Frese et al., 2019). However, due to its relative novelty, large studies have yet to use this index. This study is the first to use the DESI on a relatively large clinical scale.

The Index of Orthodontic Treatment Need Aesthetic Component (IOTN-AC) (Brook and Shaw (1989)) and the Dental Aesthetic Index (DAI) (Cons NC, 1989) are primarily used in orthodontic practice to measure and quantify dentofacial aesthetics and are not used in restorative dentistry. However, AlQarni et al. (2014) reported that the mean DAI score among young Saudi adults was 27.27 ± 13.83, indicating definite malocclusions, but no optional orthodontic treatments were needed, according to Cons et al. (1989). Al-Hummayani and Taibah (2018) reported the IOTN-AC values among Saudi young adults in Jeddah and had similar results, with 66 % of their sample having a slight or no need for orthodontic treatment according to the IOTN-AC (Brook and Shaw (1989)). These results are similar to the results of this study, with 85.3 % of the patients having good dentofacial aesthetics, according to the DESI. In the literature review, it was found that there were no studies that used the DESI, PEI, or SEI to measure dentofacial aesthetics in a Saudi population, and this study was the first to do so.

Previous studies have measured changes in the smile in the context of aging and described a decrease in maxillary incisor display during smiling and a slight increase in mandibular incisor display. However, statistical tests were not performed on most of these results (Dickens et al., 2002, Dong et al., 1999, Al Wazzan, 2004, Vig and Brundo, 1978). The findings of this study show that age negatively impacted patients' dentofacial aesthetics, and DESI scores were higher in patients over 35 years of age (*p* = 0.015), indicating the effect of age on DESI scores.

Females have been shown to be more critical in their perception of dental aesthetics than males (Zaugg et al. (2022)). Sex and age exhibit discernible effects on the morphological characteristics of anterior teeth and the curvature of the upper lip. In contrast, sex and age factors do not significantly influence the alignment of dental and facial midlines (Cunha et al. (2023)). This study shows that sex did not affect DESI scores.

The fundamental objective of esthetic restoration is to accomplish the best appearance of the teeth and maintain their vitality and function; however, the findings of this study show that direct or indirect anterior restorations negatively impacted patients' dentofacial aesthetics. Patients who had restorations in their anterior dentition scored higher on the index indicating poorer aesthetics (*p* = 0.016).

A recent systematic review addressed the impact of orthodontic treatments on smile attractiveness, revealing limited evidence supporting a moderately positive influence on smile aesthetics (Coppola et al. (2023)). Due to the limited number of patients with a history of these procedures in this study, the history of teeth bleaching and orthodontic treatments had no statistical significance.

This was the first study of its kind to use the DESI in a large clinical setting, but further epidemiological studies are recommended to investigate the sensitivity and specificity of this novel tool. Furthermore, only a single examiner recorded the findings of this study, so future studies are recommended to have multiple examiners to test the inter-rater reliability of the index.

## 5 Conclusions

This was the first study to use the DESI in a large clinical setting. Most of the patients attending Qassim University dental clinics had good dentofacial aesthetics, according to the DESI. Age and the presence of anterior restorations negatively impacted patients' dentofacial aesthetics. Sex, the nature of the chief complaint, and a history of orthodontic treatment or teeth bleaching did not affect the DESI scores. Further epidemiological studies are recommended to explore the capabilities of the DESI.

## CRediT authorship contribution statement

**Norah M Almufadhi:** Conceptualization, Data curation, Writing – original draft, Investigation, Validation, Formal analysis, Methodology, Project administration, Software. **Ola.M. Sakr:** Conceptualization, Supervision, Writing – review & editing, Investigation, Validation, Methodology. **Lina Aloufi:** Writing – review & editing, Resources, Investigation, Validation, Formal analysis, Methodology.

## Declaration of Competing Interest

The authors declare that they have no known competing financial interests or personal relationships that could have appeared to influence the work reported in this paper.

## References

[b0005] Abu Alhaija E.S.J., Al-Nimri K.S., Al-Khateeb S.N. (2005). Self-perception of malocclusion among north Jordanian school children. European Journal of Orthodontics.

[b0010] Al Wazzan K.A. (2004). The Visible Portion of Anterior Teeth at Rest. The Journal of Contemporary Dental Practice.

[b0015] Al-Hummayani F.M., Taibah S.M. (2018). Orthodontic treatment needs in saudi young adults and manpower requirements. Saudi Medical Journal.

[b0020] AlQarni M.A., Almnea R.A., Asiri W.S., Alhendi K.D., AlQahtani N.A. (2014). Evaluation of smile line in relation to age among Saudi population in Asser region. *World*. Journal of Dentistry.

[b0025] Belser U.C., Grütter L., Vailati F., Bornstein M.M., Weber H., Buser D. (2009). Outcome Evaluation of Early Placed Maxillary Anterior Single-Tooth Implants Using Objective Esthetic Criteria: A Cross-Sectional, Retrospective Study in 45 Patients With a 2- to 4-Year Follow-Up Using Pink and White Esthetic Scores. Journal of Periodontology.

[b0030] Brook P.H., Shaw W.C. (1989). The development of an index of orthodontic treatment priority. European Journal of Orthodontics.

[b0035] Cons N.C., Jenny J., Kohout F.J., DDS Y.S., Jotikastira D. (1989). Utility of the Dental Aesthetic Index in Industrialized and Developing Countries. Journal of Public Health Dentistry.

[b0040] Coppola G., Christopoulou I., Gkantidis N., Verna C., Pandis N., Kanavakis G. (2023). The effect of orthodontic treatment on smile attractiveness: a systematic review. In. Progress in Orthodontics.

[b0045] Cunha J., Fernandes G.V.O., Fernandes J.C.H., Lopes P.C., Rio R. (2023). The Interference of Age and Gender on Smile Characterization Analyzed on Six Parameters: A Clinical-Photographic Pilot Study. Medicina (lithuania).

[b0050] Dickens S.T., Sarver D.M., P. w. (2002). Changes in frontal soft tissue dimensions of the lower face by age and gender. *World*. Journal of Orthodontics.

[b0055] Dong J.-K., Jin T.H., Cho H.-W., Oh S.-C. (1999). The esthetics of the smile: a review of some recent studies. The International Journal of Prosthodontics.

[b0060] Flores-Mir C., Silva E., Barriga M.I., Lagravère M.O., Major P.W. (2004). Lay Person’S Perception of Smile Aesthetics in Dental and Facial Views. Journal of Orthodontics.

[b0065] Frese C., Staehle H.J., Wolff D. (2012). The assessment of dentofacial esthetics in restorative dentistry: A review of the literature. Journal of the American Dental Association.

[b0070] Frese C., Leciejewski F., Specht R., Wohlrab T., Büsch C., Boemicke W., Probst K., Katsikogianni E.N., Wolff D. (2019). The dental esthetic screening index: A new tool for assessment of dento-facial esthetics in restorative dentistry. Journal of Esthetic and Restorative Dentistry.

[b0075] Fürhauser R., Florescu D., Benesch T., Haas R., Mailath G., Watzek G. (2005). Evaluation of soft tissue around single-tooth implant crowns: The pink esthetic score. Clinical Oral Implants Research.

[b0080] Hickel R., Roulet J.F., Bayne S., Heintze S.D., Mjör I.A., Peters M., Rousson V., Randall R., Schmalz G., Tyas M., Vanherle G. (2007). Recommendations for conducting controlled clinical studies of dental restorative materials. In. Clinical Oral Investigations.

[b0085] Hickel R., Peschke A., Tyas M., Mjör I., Bayne S., Peters M., Hiller K.A., Randall R., Vanherle G., Heintze S.D. (2010). FDI World Dental Federation: Clinical criteria for the evaluation of direct and indirect restorations-update and clinical examples. Clinical Oral Investigations.

[b0090] Katiyar S., Gandhi S., Sodawala J., Anita G., Hamdani S., Jain S. (2016). Influence of symmetric and asymmetric alterations of maxillary canine gingival margin on the perception of smile esthetics among orthodontists, dentists, and laypersons. Indian Journal of Dental Research.

[b0095] Machado A.W., McComb R.W., Moon W., Gandini L.G. (2013). Influence of the vertical position of maxillary central incisors on the perception of smile esthetics among orthodontists and laypersons. Journal of Esthetic and Restorative Dentistry.

[b0100] Mursid S., Maharani D.A., Kusdhany L. (2020). Measuring Patient’s Orofacial Estheticsin in Prosthodontics: A Scoping Review of a Current Instrument. The Open Dentistry Journal.

[b0105] Øzhayat E.B. (2017). Responsiveness of the Prosthetic Esthetic Scale. Clinical Oral Investigations.

[b0110] Özhayat E.B., Dannemand K. (2014). Validation of the Prosthetic Esthetic Index. Clinical Oral Investigations.

[b0115] Pinzan-Vercelino C.R.M., Costa A.C.S., Ferreira M.C., Bramante F.S., Fialho M.P.N., de Gurgel J., A. (2020). Comparison of gingival display in smile attractiveness among restorative dentists, orthodontists, prosthodontists, periodontists, and laypeople. Journal of Prosthetic Dentistry.

[b0120] Prica N., Kovačić I., Čelebić A., Puhar I., Petričević N. (2022). In Middle East International Conference on Contemporary Scientific Studies.

[b0125] Roden-Johnson D., Gallerano R., English J. (2005). The effects of buccal corridor spaces and arch form on smile esthetics. American Journal of Orthodontics and Dentofacial Orthopedics.

[b0130] Rosenstiel S.F., Pappas M., Teresa Pulido M., Rashid R.G. (2009). Quantification of the esthetics of dentists’ before and after photographs. Journal of Dentistry.

[b0135] Rotundo R., Nieri M., Bonaccini D., Mori M., Lamberti E., Massironi D., Giachetti L., Franchi L., Venezia P., Cavalcanti R., Bondi E., Farneti M., Pinchi V., Buti J. (2015). The Smile Esthetic Index (SEI): A method to measure the esthetics of the smile. An intra-rater and inter-rater agreement study. European Journal of. Oral Implantology.

[b0140] Sharma P.K., Sharma P. (2012). Dental Smile Esthetics: The Assessment and Creation of the Ideal Smile. Seminars in Orthodontics.

[b0145] Tettamanti S., Millen C., Gavric J., Buser D., Belser U.C., Brägger U., Wittneben J.G. (2016). Esthetic Evaluation of Implant Crowns and Peri-Implant Soft Tissue in the Anterior Maxilla: Comparison and Reproducibility of Three Different Indices. Clinical Implant Dentistry and Related Research.

[b0150] Vig R.G., Brundo G.C. (1978). The kinetics of anterior tooth display. The Journal of Prosthetic Dentistry.

[b0155] Zaugg F.L., Molinero-Mourelle P., Abou-Ayash S., Schimmel M., Brägger U., Wittneben J.G. (2022). The influence of age and gender on perception of orofacial esthetics among laypersons in Switzerland. Journal of Esthetic and Restorative Dentistry.

